# Socio-demographic and AIDS-related factors associated with tuberculosis stigma in southern Thailand: a quantitative, cross-sectional study of stigma among patients with TB and healthy community members

**DOI:** 10.1186/1471-2458-11-675

**Published:** 2011-08-30

**Authors:** Aaron M Kipp, Petchawan Pungrassami, Kittikorn Nilmanat, Sohini Sengupta, Charles Poole, Ronald P Strauss, Virasakdi Chongsuvivatwong, Annelies Van Rie

**Affiliations:** 1 University of North Carolina, Department of Epidemiology, Chapel Hill, North Carolina, USA; 2 Tuberculosis Centre, Region 12 Yala, Thailand; 3 Prince of Songkla University, Department of Medical Nursing, Hat Yai, Thailand; 4 University of North Carolina, Department of Social Medicine, Chapel Hill, North Carolina, USA; 5 University of North Carolina, Department of Dental Ecology, Chapel Hill, North Carolina, USA; 6 Prince of Songkla University, Epidemiology Unit, Hat Yai, Thailand

## Abstract

**Background:**

Tuberculosis (TB) remains one of the most important infectious diseases worldwide. A comprehensive approach towards disease control that addresses social factors including stigma is now advocated. Patients with TB report fears of isolation and rejection that may lead to delays in seeking care and could affect treatment adherence. Qualitative studies have identified socio-demographic, TB knowledge, and clinical determinants of TB stigma, but only one prior study has quantified these associations using formally developed and validated stigma scales. The purpose of this study was to measure TB stigma and identify factors associated with TB stigma among patients and healthy community members.

**Methods:**

A cross-sectional study was performed in southern Thailand among two different groups of participants: 480 patients with TB and 300 healthy community members. Data were collected on socio-demographic characteristics, TB knowledge, and clinical factors. Scales measuring perceived TB stigma, experienced/felt TB stigma, and perceived AIDS stigma were administered to patients with TB. Community members responded to a community TB stigma and community AIDS stigma scale, which contained the same items as the perceived stigma scales given to patients. Stigma scores could range from zero to 30, 33, or 36 depending on the scale. Three separate multivariable linear regressions were performed among patients with TB (perceived and experience/felt stigma) and community members (community stigma) to determine which factors were associated with higher mean TB stigma scores.

**Results:**

Only low level of education, belief that TB increases the chance of getting AIDS, and AIDS stigma were associated with higher TB stigma scores in all three analyses. Co-infection with HIV was associated with higher TB stigma among patients. All differences in mean stigma scores between index and referent levels of each factor were less than two points, except for incorrectly believing that TB increases the chance of getting AIDS (mean difference of 2.16; 95% CI: 1.38, 2.94) and knowing someone who died from TB (mean difference of 2.59; 95% CI: 0.96, 4.22).

**Conclusion:**

These results suggest that approaches addressing the dual TB/HIV epidemic may be needed to combat TB stigma and that simply correcting misconceptions about TB may have limited effects.

## Background

Tuberculosis (TB) remains one of the most important infectious diseases worldwide with an estimated 9.4 million new cases and 1.7 million deaths in 2009 [1]. In addition to the focus on treatment and cure rates, a more comprehensive approach towards disease control is now advocated, in order to address poverty and other social risk factors, of which stigma is one component [2]. Stigma was originally described by Goffman as "an undesirable or discrediting attribute that an individual possesses, thus reducing that individual's status in the eyes of society" [3]. More recently, health related stigma has been defined as "a social process or related personal experience characterized by exclusion, rejection, blame, or devaluation that results from experience or reasonable anticipation of an adverse social judgment about a person or group identified with a particular health problem" [4]. This conceptualization highlights two important components of stigma: overt patient experiences of discrimination in their community and the internalized fear and anticipation of social consequences regardless of any actual experience by the patient. Defining stigma as a "social process" draws attention to the important interactions between those who have the "health problem" (e.g. patients with TB) and those who do not (e.g. healthy community members)[5].

Individuals diagnosed with TB report fears of isolation and rejection such as losing employment, being divorced or having diminished marriage prospects, not being allowed to share meals, utensils or sleeping quarters with family members, and general avoidance or gossip among community members [6-9]. Fear of these consequences may lead to delays in seeking care for TB symptoms and could affect adherence to treatment [6,7,10]. Because of this, TB stigma continues to be viewed as a barrier to TB control.

Qualitative studies using focus groups and interviews have identified religion, socioeconomic status, level of education, and gender as possible factors associated with TB stigma [11,12], with women more often than men feeling the effects of TB stigma [6,8]. Reasons why TB may be stigmatizing include contagiousness [7,13,14], incorrect knowledge of its cause, transmission, or treatment [7,8,12,13,15], or its association with marginalized groups such as the poor, ethnic minorities, sex workers, prisoners, and those infected with HIV [7,8,12,13,16,17]. As the epidemics of TB and HIV/AIDS have converged in many areas of the world, there is growing concern that AIDS and AIDS stigma may compound existing TB stigma [13,18,19]. For some, a TB diagnosis is thought to be synonymous with an AIDS diagnosis, or TB symptoms may be misidentified as AIDS symptoms [12,14,18-20].

While qualitative studies help identify possible factors associated with stigma, quantitative measures are needed to assess the level of stigma in a population, factors most strongly associated with stigma, and evaluate the effectiveness of interventions [4]. Unfortunately, few quantitative studies have been done to assess factors associated with TB stigma [21]. Except for one study [22], previous quantitative studies used brief scales that were not formally developed or not evaluated for internal consistency [23,24]. Furthermore, each of these quantitative studies measured TB stigma among patients only. Given the lack of empirical studies on TB stigma, it cannot be assumed that stigma levels and associated factors in patients with TB are identical to the stigma levels in the community and the factors associated with community stigma.

We used formally developed and validated scales [25] to measure the level of TB stigma among patients with TB and healthy community members. The primary purpose of this study was to test if socio-demographic characteristics, TB knowledge, TB clinical factors, and AIDS stigma were associated with higher TB stigma among two different populations: patients with TB and healthy community members. Identification of factors that affect stigma, particularly among both groups, would help inform interventions at the patient, provider, organizational, and community level.

## Methods

### Study site and participants

Data for this analysis come from a larger study to measure TB and AIDS stigma in southern Thailand [12,25] and identify its effects on care and treatment [26,27]. Adults (> 17 years old) with TB were enrolled between August 2005 and July 2006 at the regional TB center or one of seven hospital-based TB clinics (six public and one private). Patients were eligible if they had been receiving TB treatment for less than one month and did not have a prior history of TB treatment. In April 2007 a convenience sample of healthy, adult community members were invited to participate while visiting friends or family members (admitted for reasons other than TB or AIDS) at two of the study hospitals. To rule out TB disease among community participants, individuals were not eligible if they reported a cough for three weeks or longer.

This study was approved by the Institutional Review Boards of the University of North Carolina and the Prince of Songkla University. Written informed consent was obtained from patients, while community members provided oral consent.

### Stigma measurement

The TB and AIDS stigma scales were developed using input from patients with TB, patients with AIDS, family members, and community members and were validated in a population of patients with TB [12,25]. For this analysis, TB and AIDS stigma were measured among patients with TB using three of these scales: experienced/felt TB stigma (12 items) measures the experiences, thoughts and feelings of patients with TB (e.g. "Some people who have TB lose friends when they share with them they have TB"; "Some people who have TB feel alone"); perceived TB stigma (11 items) measures the patients' perception of how community members feel about or act toward people who have TB (e.g. "Some people try not to touch others with TB"; "Some people are afraid of those with TB"); perceived AIDS stigma (10 items) measures the patient with TB's perception of how community members feel about or act toward people who have AIDS.

We performed a factor analysis to determine if the items assessing perceived TB and AIDS stigma in patients (e.g."Some people try not to touch others with TB") were also valid for use in community members. Results showed that all 11 TB stigma items loaded on a single factor with item factor loadings between 0.45 and 0.70. All but one of the original 11 AIDS stigma items loaded on a single factor with item factor loadings between 0.40 and 0.78. The item "Some people think that people with AIDS get what they deserve" had a factor loading < 0.30 and was deleted from all analyses. When administered to community members, these scales are referred to as community TB and community AIDS stigma scales because they measure how people in the community feel about or act toward people who have TB or AIDS. As a result, they compliment the perceived TB and AIDS stigma scales which ask about the patients' perception of stigma in the community.

Stigma items were scored on a Likert scale with four levels: strongly disagree (0), disagree (1), agree (2), and strongly agree (3). Responses were summed for each scale to create stigma scores, with higher responses indicating higher stigma. Internal reliability was high for all four scales, ranging from 0.82 to 0.93. Scores on the experienced and felt TB stigma scale could range from 0 to 36, scores on the perceived TB and perceived AIDS stigma scales could range from 0 to 33, and scores on the community TB and community AIDS scales could rand from 0 to 30.

### Factors potentially associated with TB stigma

Based on review of the published literature, age, gender, religion, socioeconomic status, TB knowledge, TB symptoms (patients only), self-reported HIV status (patients only), and knowing someone with TB (community members only) were considered as potential factors associated with TB stigma. Information on these factors was collected by trained interviewers using a questionnaire. Information on knowing someone with TB was not collected from patients with TB. Information on HIV status was not collected for community members in an effort to increase participation. The adult prevalence of HIV in southern Thailand is low (0.19% among blood donors and 0.59% among women at ante-natal clinics) [28], which suggests that community members were likely HIV uninfected.

### Analysis

For patients with TB, perceived TB and perceived AIDS stigma scores were standardized to the age (10 year categories), gender, religion, and education distribution of the community members to allow some comparison of stigma levels reported by patients with TB and community members.

Confounders of the relationship between each factor potentially associated with TB stigma and TB stigma scores were identified using causal diagrams [29,30]. Causal diagrams show the expected or assumed causal relationships between the independent and dependent variables and other covariates. They make explicit the *a priori *causal knowledge of the investigator necessary to identify confounders and do not rely on data-driven or statistical approaches to confounding. Using a series of graphical rules to assess whether two variables are marginally (in)dependent, a set of adjustment variables sufficient to control for confounding by measured covariates can be identified. While the end result is often similar to data driven approaches, it avoids some shortcomings of those methods. Continuous TB stigma scores were modeled as separate outcomes using multivariable linear regression (SAS 9.1, PROC GENMOD with identity link and normal distribution), with separate regression models built for each potential determinant, first among patients with TB, then among community members (see footnote in Table 1 for the sets of confounders used in each multivariable analysis). Model coefficients are interpreted as the mean difference in stigma scores between the index and referent categories for each factor of interest (socio-demographic characteristics, TB knowledge, clinical, and AIDS stigma).

**Table 1:** Adjusted differences in mean, summed stigma scores for participant characteristics

		**Patients with TB**						**Community members**		
			
		**Perceived TB stigma**			**Experienced/felt TB stigma**			**Community TB stigma**		
			
		Crude*	**MD†**	**(95% CI)**	Crude*	**MD†**	**(95% CI)**	Crude*	**MD†**	**(95% CI)**
			
Age	Age (per 10 year)	18.12	0.61	(0.29, 0.92)	19.72	0.17	(-0.12, 0.45)	19.96	0.66	(0.22, 1.11)
	
Gender	Male	18.51	0.0		20.16	0.0		19.88	0.0	
	Female	18.28	0.16	(-0.79, 1.11)	19.28	-0.80	(-1.67, 0.07)	20.37	0.30	(-0.80, 1.39)
	
Religion	Buddhist	18.88	0.0		19.84	0.0		20.02	0.0	
	Muslim	17.51	-1.37	(-2.32, -0.42)	19.87	0.04	(-0.83, 0.90)	20.59	0.56	(-0.85, 1.98)
	
	Completed secondary	18.44	0.0		19.16	0.0		19.71	0.0	
Education	Completed primary	17.89	-0.41	(-1.53, 0.72)	19.78	0.54	(-0.46, 1.55)	19.95	0.21	(-1.03, 1.46)
	Some or no primary	19.08	0.78	(-0.39, 1.96)	20.52	1.22	(0.17, 2.28)	21.38	1.61	(0.14, 3.08)
	
Income	Baht/month (1,000 baht)	18.38	0.00	(-0.04, 0.03)	19.39	-0.02	(-0.05, 0.02)	19.39	-0.01	(-0.06, 0.04)
	
	Infection from others	18.96	0.0		19.77	0.0		19.81	0.0	
	Eat/drink with patient	17.58	-1.46	(-3.87, 0.95)	19.20	-0.62	(-2.76, 1.52)	20.42	1.13	(-0.86, 3.11)
TB Knowledge	Smoking/drinking	18.62	-0.66	(-1.97, 0.65)	20.49	0.42	(-0.77, 1.62)	20.09	0.03	(-1.57, 1.63)
(Cause)	Work hard	17.91	-1.57	(-3.15, 0.01)	19.97	-0.17	(-1.61, 1.28)	24.00		Too few
	Heredity	17.79	-0.98	(-3.40, 1.44)	18.65	-1.23	(-3.37, 0.92)	19.44	-1.44	(-3.78, 0.91)
	Weak body	18.80	-0.30	(-1.78, 1.17)	19.44	-0.36	(-1.71, 0.99)	20.04	0.43	(-1.84, 2.69)
	
	Not cough or sneeze/don't know	18.43	0.0		19.67	0.0		20.25	0.0	
	Cough/sneeze	18.43	0.08	(-1.09, 1.25)	19.89	0.40	(-0.67, 1.47)	20.09	-0.28	(-1.51, 0.95)
	Not eat or drink/don't know	18.15	0.0		19.61	0.0		19.41	0.0	
TB Knowledge	Eat/drink	18.61	0.52	(-0.39, 1.43)	20.01	0.44	(-0.39, 1.26)	21.39	1.73	(0.62, 2.84)
(Transmission)	Not touch/don't know	18.43	0.0		19.74	0.0		20.12	0.0	
	Touch	18.49	-0.14	(-1.73, 1.45)	21.00	1.21	(-0.22, 2.64)	20.22	-0.33	(-2.17, 1.51)
	Not set/don't know	18.33	0.0		19.75	0.0		20.05	0.0	
	Sex	19.35	1.06	(-0.44, 2.55)	20.84	0.92	(-0.46, 2.30)	21.63	1.94	(-0.47, 4.34)
	
TB Knowledge	Not curable	19.63	0.0		20.67	0.0		20.65	0.0	
(Cure)	Curable	18.39	-1.41	(-3.84, 1.02)	19.83	-0.91	(-3.17, 1.35)	19.97	-0.93	(-2.23, 0.37)
	
	TB does not increase AIDS/don't know	17.84	0.0		18.74	0.0		19.87	0.0	
	TB increases AIDS	19.02	1.08	(0.19, 1.86)	20.93	2.16	(1.38, 2.94)	20.42	0.55	(-0.52, 1.62)
TB Knowledge‡	AIDS does not increase TB/don't know	18.12	0.0		19.37	0.0		19.52	0.0	
(TB/HIV)	AIDS increases TB	18.55	0.42	(-0.59, 1.42)	20.03	0.85	(-0.07, 1.77)	20.36	0.86	(-0.35, 2.06)
	AIDS and TB do not appear similar	17.83	0.0		19.15	0.0		20.52	0.0	
	AIDS/TB appear similar	18.68	0.86	(-0.12, 1.84)	20.14	0.86	(-0.03, 1.75)	19.89	-0.55	(-1.66, 0.57)
	
HIV/AIDS stigma	Perceived/Community AIDS stigma	19.79	0.64	(0.58, 0.71)	21.79	0.48	(0.41, 0.55)	21.00	0.55	(0.45, 0.64)
	
Know person	Did not know person with TB							19.75	0.0	
with TB	Knew person with TB (lived)							19.98	0.13	(-1.32, 1.57)
	Knew person with TB (died)							22.42	2.59	(0.96, 4.22)
	
	Never tested	18.42	0.0		19.72	0.0				
HIV status^§^	Previously tested negative	18.17	0.04	(-1.05, 1.13)	19.44	-0.05	(-1.04, 1.94)			
	Previously tested positive	18.91	0.90	(-0.42, 2.22)	21.09	1.57	(0.39, 2.76)			
	
	Cough	18.70	0.0		19.98	0.0				
	Hemoptysis	18.25	-0.43	(-1.54, 0.69)	19.48	-0.57	(-1.57, 0.42)			
Symptoms§‡	Weight loss	18.57	-0.28	(-1.78, 1.22)	20.79	0.48	(-0.89, 1.85)			
	Fever and/or EPTB only	17.04	-1.61	(-3.11,-0.10)	19.17	-0.51	(-1.85, 0.83)			
	No symptoms	20.36	1.73	(-0.94, 4.40)	19.77	-0.18	(-2.64, 2.28)			

*Crude, mean stigma scores for each group. For continuous variables, mean stigma scores are reported for ages 30 to 39 years old, those with 10,000 baht income, and AIDS stigma scores of 20.

† MD, adjusted mean difference in summed stigma scores; CI, confidence interval; 0.0 indicates referent level. Sets of confounders used in multivariable analysis of each factor are as follows:

**Age and Religion**: no confounders; **Gender**: adjusted for age and religion; **Income**: adjusted for age, religion and education; **Education**: adjusted for religion and gender; **TB knowledge variables**: adjusted for age, education, income (patients only) and knowing someone with TB (community members only); **AIDS stigma**: adjusted for age, gender, religion, education, income, TB/HIV knowledge, HIV status (patients only) and TB symptoms (patients only); **Knowing someone with TB **(community members only): adjusted for age and income; **HIV status **(patients only): adjusted for age, religion and income; **TB symptoms **(patients only): adjusted for gender and HIV status

‡ Cough and hemoptysis categories may also include patients with additional symptoms such as weight loss, fever, or EPTB (extrapulmonary TB); Weight loss excludes cough but may include other symptoms (e.g. fever and/or EPTB); Fever and EPTB includes patients with only those symptoms (no cough or weight loss).

§ Self-reported

## Results

### Participant characteristics

During the study period, 1,057 adult patients with TB were registered of whom 929 (88%) were eligible for participation. Of those who were eligible, 132 (14%) were too weak or too old to communicate clearly with interviewers and were not enrolled, 317 (34%) refused participation, and the remaining 480 (52%) were enrolled. In addition, 300 healthy community members were enrolled. Compared with community members, patients tended to be older, less educated, and more often male and Muslim (Table 2). Infection, smoking, or having a weak body were common beliefs about the cause of TB among all participants. Community members also believed eating or drinking with a person who has TB is a cause of TB, whereas patients believed working hard can cause TB. Most participants stated that TB can be transmitted by a cough or sneeze, but beliefs that transmission occurs through routes such as eating and drinking with a person who has TB were also common, especially among patients. Nearly all patients and most community members knew TB was curable. High awareness of a link between TB and HIV was present among all participants.

**Table 2:** Distribution of participant characteristics

Continuous participant characteristics		Patients with TB		Community members	
Age	Age in years (median, range)	37	(18 - 79)	34	(18 - 69)
Income	Thousand Baht per month (median, range)	10	(0 - 90)	10	(1 - 100)

**Categorical participant characteristics**		**N**	**(%)**	**N**	**(%)**

Gender	Male	317	(66.0)	146	(48.7)
	Female	163	(34.0)	154	(51.3)

Religion	Buddhist	319	(66.7)	244	(81.3)
	Muslim	159	(33.3)	56	(18.7)

Education	Less than or no primary school	160	(33.4)	61	(20.3)
	Completed primary school	191	(39.8)	103	(34.3)
	Completed secondary school	129	(26.9)	136	(45.3)

TB knowledge*	Infected from family/others	87	(18.1)	42	(14.0)
(Cause)	Work hard	71	(14.8)	3	(1.0)
	Smoking/drinking	162	(33.8)	158	(52.7)
	Heredity	20	(4.2)	25	(8.3)
	Weak body	82	(17.1)	27	(9.0)
	Eat or drink with patient	20	(4.2)	43	(14.3)
	Other	38	(7.9)	2	(0.7)

TB knowledge†	Eat/drink	291	(61.9)	109	(36.7)
(Transmission)	Touch	41	(8.7)	32	(10.8)
	Sex	46	(9.8)	16	(5.4)
	Cough/sneeze	396	(84.3)	224	(75.4)
	Other	64	(13.6)	50	(16.8)

TB knowledge	Curable	463	(96.5)	231	(77.0)
(Cure)	Not curable	17	(3.5)	69	(23.0)

TB knowledge†	TB increase AIDS	242	(50.4)	142	(47.3)
(TB/HIV)	AIDS increases TB	346	(72.1)	219	(73.0)
	AIDS/TB appear similar	339	(70.6)	186	(62.0)

Knew person	Did not know person with TB			211	(70.3)
with TB	Knew person with TB (lived)			51	(17.0)
	Knew person with TB (died)			38	(12.7)

HIV status‡	Never tested	292	(60.8)		
	Previously tested negative	115	(24.0)		
	Previously tested positive	73	(15.2)		

Symptoms§	Cough	236	(49.2)		
	Hemoptysis	116	(24.2)		
	Weight loss	59	(12.3)		
	Fever and/or extrapulmonary only	55	(11.5)		
	No symptoms	14	(2.9)		

* Exclusive or † non-exclusive categories

§ Cough and hemoptysis categories may also include patients with additional symptoms such as weight loss, fever, or EPTB (extrapulmonary TB); Weight loss excludes cough but may include other symptoms (e.g. fever and/or EPTB); Fever and EPTB includes patients with only those symptoms (no cough or weight loss).

‡Self-reported

Most patients reported symptoms typically associated with TB. Upon diagnosis of TB, 73 (15.2%) patients were aware of their HIV co-infection and 115 (24%) had a negative test in the past. The majority (292; 60.8%), however, had never been tested for HIV. Among community members, 89 (29.7%) knew one or more persons who suffered from TB regardless of outcome, while 38 (12.7%) knew someone who had died from TB.

### Stigma scale responses

On the TB stigma scales, 14 (2.9%) and 20 (4.2%) patients were excluded from the perceived stigma and experienced/felt stigma scale analyses, respectively, due to missing item responses. On the perceived AIDS stigma scale, 15 (3.1%) patients had missing item responses. All community members had complete item responses. Internal consistency (Cronbach's coefficient alpha) for all three scales was good (range 0.82 to 0.91) and all scores were normally distributed with mean scores ranging from 17.20 to 20.13 (Table 3).

**Table 3:** Stigma scale characteristics and scores

	Cronbach's alpha	Mean summed stigma score (SD)	Kurtosis	Skewness
**Tuberculosis stigma**				

Patients with TB: experienced and felt TB stigma (12 items)	0.82	19.85 (4.43)	1.35	0.00
Patients with TB: perceived TB stigma (11 items)	0.88	18.43 (4.95)	0.36	-0.06
Community members: community TB stigma (11 items)	0.85	20.13 (4.87)	0.31	0.08
**AIDS stigma**				

Patients with TB: perceived AIDS stigma (10 items)	0.91	17.20 (4.88)	0.65	-0.35
Community members: community AIDS stigma (10 items)	0.87	19.14 (4.86)	0.46	-0.12

The mean community TB stigma score was higher than the mean perceived TB stigma score reported by patients (20.13 vs.18.43 or 18.25 after standardization and exclusion of patients with known HIV infection). The mean community AIDS stigma score was also higher than the mean perceived AIDS stigma score reported by patients with TB, (19.14 vs. 17.20 or 16.90 after standardization and exclusion of patients with known HIV infection). In both groups, AIDS stigma scores were only slightly higher than TB stigma scores.

### Factors associated with TB stigma

Mean differences (MD) in TB stigma scores between index and referent levels of each factor potentially associated with TB stigma were small (Table 1), with 75% of estimates having less than a one point difference in stigma score. Only two estimates were greater than a two point difference in stigma score. Believing that TB increases the chance of getting AIDS was associated with experienced/felt TB stigma among patients (MD 2.16; 95% CI: 1.38, 2.94) and knowing someone who died of TB was associated with community TB stigma among community members (2.59; 95% CI: 0.96, 4.22).

*Factors associated with TB stigma among patients with TB*. Few factors had consistent MDs in stigma scores for both the perceived TB stigma and experienced/felt TB stigma scales.. Older age (0.66; 0.22, 1.11 per 10 year increase) and Muslim religion (-1.37; 95% CI: -2.32, -0.42) were both associated with perceived TB stigma, while being female (-0.80; 95% CI: -1.67, 0.07) was only associated with experienced/felt TB stigma. Patients with some or no primary education reported higher scores on both TB stigma scales, especially the experienced/felt stigma scale (1.22; 95% CI: 0.17, 2.28), compared to patients who completed secondary education. Patients who reported incorrect beliefs about the cause of TB consistently had lower scores on both TB stigma scales than patients who knew that infection was the cause of TB, but all estimates were imprecise. There was also no association between knowledge about TB transmission or believing TB was curable and TB stigma.

Knowledge and clinical factors related to HIV/AIDS tended to be associated with higher TB stigma. Correctly knowing that AIDS increases the likelihood of developing TB and that both AIDS and TB symptoms appear similar was associated with slightly higher scores on both TB stigma scales. Patients who believed that having TB could increase the likelihood of having AIDS reported much higher scores on the perceived TB stigma scale (1.08; 95% CI: 0.19, 1.86) and the experienced/felt TB stigma scale (2.16; 95% CI: 1.38, 2.94). Similarly, patients reporting higher perceived AIDS stigma also had higher scores on the perceived TB stigma scale (0.64; 95% CI: 0.58, 0.71 per one point increase in the AIDS stigma score) and the experienced/felt TB stigma scale (0.48; 95% CI: 0.41, 0.55). Patients who were aware of their HIV co-infection prior to study enrollment had higher stigma scores, especially on the experienced/felt TB stigma scale (1.57; 95% CI: 0.39, 2.76) compared to those who had never been tested. In contrast, patients presenting with fever only or extrapulmonary symptoms, presentations which are more common among HIV-infected patients with TB, had lower stigma, especially on the perceived TB stigma scale (-1.61; 95% CI: -3.11, -0.10).

*Factors associated with TB stigma among community members*. Similar to patients with TB, both older age (0.66; 95% CI: 0.22, 1.11) and having some or no primary education (1.61; 95% CI: 0.14, 3.08) were associated with higher community TB stigma scores. Knowledge of TB did not show any association with community TB stigma, except for believing that TB can be transmitted though eating and drinking with a patient (1.73; 95% CI: 0.62, 2.84). Unlike the results for patients with TB, there was no association between TB/HIV knowledge and TB stigma. Reporting higher community AIDS stigma, however, was associated with higher community TB stigma (0.55; 95% CI: 0.45, 0.64 per one point increase). Knowing someone who died of TB was also associated with higher community TB stigma (2.59; 95% CI: 0.96, 4.22) compared with not knowing anyone with TB.

## Discussion

Most factors analyzed in this study had only minimal or inconsistent associations with TB stigma. Only poor education (some or no primary education) and AIDS stigma showed a consistent association with higher TB stigma in all three analyses. In addition to low levels of formal education, the largest, most precise, and consistent associations with higher TB stigma were beliefs relating to the intersection of TB with HIV, HIV co-infection, and the level of AIDS stigma. Knowing someone who died of TB had the largest association with TB stigma. Individuals in areas affected by HIV are more likely to believe that TB is not curable [13,22]. Thus, higher TB stigma due to knowing someone who has died of TB may be linked to HIV co-infection via the belief that TB is not curable, a result that was observed, but not statistically supported, in our study.

Most prior studies on TB stigma have been qualitative. A qualitative study in the same area of southern Thailand found that religion, low TB knowledge, severe symptoms, and symptoms similar to AIDS were related to stigmatizing attitudes towards TB [12]. While some of these qualitative observations were confirmed by our quantitative findings, most factors identified as being associated with TB stigma in the qualitative study showed small, inconsistent or imprecise effects in our study. Only among community members was the belief that eating and drinking with a patient could transmit TB found to be associated with a larger increase in stigma. These studies underscore the importance of quantitative analyses to assess the generalizability and magnitude of associations observed in qualitative studies.

To date, only three other quantitative studies of TB stigma and potential factors associated with TB stigma have been published [22-24]. Detailed comparisons between them warrant caution, as different scales, study populations, and potential factors were used. Similar to our findings, however, lack of consistent associations between factors and stigma among different populations was observed by Somma et al.[22] from a study in Bangladesh, India, Malawi and Colombia. In that study, only marital status, financial problems, social distress, and seeking care at a private hospital were found to increase stigma in more than one study site.

Documenting the level of TB stigma and identifying factors associated with stigma are important steps towards developing interventions to reduce stigma [4]. It is generally believed that increasing TB knowledge and education are important interventions to decrease stigma [8,31]. We found, however, that TB knowledge itself has a minor and inconsistent association with TB stigma. We did find that incorrectly believing TB can increase the chance of getting AIDS was associated with higher stigma among patients. This is most likely due to the rate of TB/HIV co-infection and/or that patients with TB are routinely tested for HIV. Thus, having TB increases the chances of *being diagnosed with*, but not being infected with, HIV. In our study, belief that TB increases the chance of getting AIDS, AIDS stigma, co-infection with HIV, and knowing someone who died from TB had the strongest, most precise, and most consistent association with higher TB stigma. This is consistent with the suggestion that AIDS and AIDS stigma may compound existing TB stigma [13,18,19] and suggests that interventions aimed at decreasing TB stigma should also address the HIV/AIDS epidemic. This may include media and public education campaigns to raise awareness that TB occurs and is curable, both in HIV-infected individuals and those not infected with HIV. This could coincide with the message that people living with HIV should be watchful for symptoms of TB and seek medical care if they develop common TB symptoms, with making HAART available to all patients needing treatment for HIV, and providing isoniazid preventive therapy (IPT) to patients with HIV. It has been suggested that scaling up the provision of HAART may decrease AIDS stigma by reducing AIDS mortality and allowing for normalization of patients [32-34], while provision of IPT would reduce the burden of HIV-related TB morbidity and mortality, thereby changing the perception that patients with HIV also develop TB [35-37].

Some limitations of our study should be noted. First, the small number of responses for some causes and transmission routes of TB could have led to low power to detect a true association with stigma. Second, it is possible that associations were biased as high levels of stigma may have caused patients to avoid seeking any care for their symptoms, high levels of stigma may have been the reason why some eligible patients refused to participate in the study, or a period of coping could have occurred between the onset of symptoms and presentation at the clinic, resulting in lower levels of stigma. This could explain, in part, why patients with TB reported lower TB and AIDS stigma than community members, because there is no reason to think any selection bias occurred among community members. Finally, we did not assess factors associated with stigma during TB treatment.

## Conclusion

Few quantitative studies on TB stigma and potential factors associated with TB stigma have been published. We report the presence of TB stigma in both patients and community members in southern Thailand. Our results suggest that interventions addressing the dual TB/HIV epidemic may be needed to combat TB stigma, and that simply improving knowledge about TB may have limited effects.

## Competing interests

The authors declare that they have no competing interests.

## Authors' contributions

AMK is responsible for data analysis and writing the manuscript. PP and KN are responsible for acquisition of data. SS assisted with study conception, design, and analysis. CP assisted with data analysis and interpretation. AMK, RPS, VC, and AVR are responsible for study conception and design. All authors contributed critical reviews of the manuscript and have read and approved the final manuscript.

## Pre-publication history

The pre-publication history for this paper can be accessed here:

http://www.biomedcentral.com/1471-2458/11/675/prepub
